# ZMIZ1 Regulates Proliferation, Autophagy and Apoptosis of Colon Cancer Cells by Mediating Ubiquitin–Proteasome Degradation of SIRT1

**DOI:** 10.1007/s10528-023-10573-9

**Published:** 2024-01-12

**Authors:** Min Huang, Junfeng Wang, Zhengrong Zhang, Xueliang Zuo

**Affiliations:** Department of Gastrointestinal Surgery, The First Affiliated Hospital of Wannan Medical College, No.2 Zheshan West Road, Wuhu, 241000 Anhui China

**Keywords:** Colon cancer, Proliferation, Autophagy, Apoptosis, ZMIZ1, STIR1, FOXO3a

## Abstract

There are nearly 1.15 million new cases of colon cancer, as well as 586,858 deaths from colon cancer worldwide in 2020. The aim of this study is to reveal whether ZMIZ1 can control the fate of colon cancer cells and the mechanism by which it functions. Specific shRNA transfection was used to knock down the expression of ZMIZ1 in colon cancer cell lines (HCT116 and HT29), and cell proliferation was detected using EdU and CCK-8 reagents, apoptosis by flow cytometry, and autophagy by western blot. The interaction of ZMIZ1 and SIRT1 was analyzed. Knockdown of ZMIZ1 significantly inhibited autophagy and proliferation, and induced apoptosis of HCT116 and HT29 cells. The mRNA level of SIRT1 was not affected by ZMIZ1 knockdown, but the protein level of SIRT1 was significantly decreased and the protein level of the SIRT1-specific substrate, acetylated FOXO3a, was reduced. Immunoprecipitation assays identified the interaction between SIRT1 and ZMIZ1 in HCT116 and HT29 cells. ZMIZ1 increased intracellular ubiquitination of SIRT1. Knockdown or pharmacological inhibition of SIRT1 neutralized the effects of ZMIZ knockdown on proliferation, autophagy and apoptosis in HCT116 and HT29 cells. ZMIZ1 may control the fate of colon cancer cells through the SIRT1/FOXO3a axis. Targeting ZMIZ1 would be beneficial for the treatment of colon cancer.

## Introduction

According to GLOBOCAN's global cancer estimates, there are close to 1.15 million new cases of colon cancer, as well as 586,858 deaths from colon cancer in 2020 (Sung et al. 2021). Furthermore, it is predicted that, colon cancer deaths will increase significantly in all countries by 2035 due to population growth and aging, despite heterogeneous trends in incidence and mortality between countries and regions (Araghi et al. 2019). It remains critical to explore new molecules that can determine colon cancer cell fate and play a key role in colon cancer progression, as well as may be available as therapeutic targets.

The single nucleotide polymorphisms (SNPs) in the *ZMIZ1* gene have been identified by several genome-wide association studies (GWAS) to be associated with the risk of prostate cancer (Takata et al. 2019; Zhang et al. 2022), colon cancer (Arnau-Collell et al. 2020; Song et al. 2017; Zhang et al. 2014) and breast cancer (O'Brien et al. 2014). Furthermore, a study by Mathios et al*.* indicates that the methylation status of the *ZMIZ1* gene predicts the outcome of a variety of solid cancers, including glioblastoma, astrocytoma, bladder cancer, and renal cell carcinoma, of which patients with hypomethylation of the *ZMIZ1* gene exhibit worse survival compared to patients with hypermethylation of the gene (Mathios et al. 2019). Hypomethylation of the gene usually predicts high transcription and expression of the gene. ZMIZ expression is also considered to be a marker for a variety of cancers (Bhadra et al. 2022). ZMIZ1 is overexpressed in breast and colon cancers, of which patients exhibit reduced survival with high expression levels of ZMIZ1 (Mathios et al. 2019). These cohort studies suggest that the expression of ZMIZ1 is closely associated with cancer progression, and further affects patient prognosis. However, molecular experiments are still lacking. The specific molecular functions of ZMIZ1, as well as its specific mechanisms involved in cancer progression remain completely unknown.

Sirtuin 1 (SIRT1) is a conserved mammalian NAD-dependent histone/protein deacetylase that plays a key role in cellular metabolism, stress response, genomic stability and aging. Recent studies have reported its role in colon cancer, either as a tumor suppressor or as a tumor promoter. Both its expression pattern in colon cancer tissues and relevance to patient survival (Jung et al. 2013; Kriegl et al. 2012; Song et al. 2018), and the effects of knockdown, overexpression or pharmacological inhibition on the behavior of cancer cells (Ghosh et al. 2017; Lee et al. 2022; Song et al. 2014) have been reported with conflicting results, which are currently difficult to reconcile. The role of SIRT1 in the multistep process of carcinogenesis is extremely complex.

In this study, we will knock down the expression of ZMIZ1 in colon cancer cells in vitro, and examine its effect on cancer cell fate decisions, as well as reveal the molecular function of ZMIZ1 as a E3 ubiquitin ligase that promotes colon cancer progression by mediating SIRT1 ubiquitination degradation.

## Materials and Methods

### Cell Culture, Transfection and Treatment

Human colon cancer cell lines (HCT116 and HT29) were obtained from the American Type Culture Collection (ATCC), and cultured in Dulbecco’s Modified Eagle’s Medium (KGM12800N-500, KeyGEN BioTECH) supplemented with 10% FBS (A31608, Gibco), 100 g/mL penicillin–streptomycin (KGY0023, KeyGEN BioTECH) in an incubator with 5% CO_2_ at 37 °C. The shRNA-ZMIZ1 was designed and synthesized by MERKE (TRCN0000017413) with the following sequence: 5’-CCAGGCGTATAACAGCCAATT-3’, and pLKO.1-puro empty vector (SHC001, Sigma-Aldrich) was used as a control. shRNA was transiently transfected into cells using Lipofectamine 2000 (11,668,019, Invitrogen) in accordance with the manufacturer’s instructions. SIRT1 selective inhibitor Selisistat, also known as EX-527 (HY-15452), was purchased from MedChemExpress, and 100 nM of EX-527 was added to the medium to treat the cells.

### Western Blot

Cells were lysed on ice using pre-chilled RIPA lysis buffer (CW2333S, CWBIO) containing protease inhibitors (CW2200S, CWBIO). Cell lysates were centrifuged at 12,000 rpm for 10 min at 4 °C, and the supernatant protein samples were collected. Protein concentration was determined using the BCA Protein Assay Kit (CW0014S, CWBIO). Aliquots of protein samples were heat denatured in a water bath, separated by SDS-PAGE electrophoresis on the basis of molecular weight, and subsequently electrotransferred onto a PVDF membrane (0.45 μm, IPVH00010; 0.2 μm, ISEQ00010; Millipore). After being blocked with 5% skim milk for 1 h at room temperature, the membrane was sequentially incubated with primary antibodies overnight at 4 °C and secondary antibodies for 2 h at room temperature. Finally, the bands were visualized using the Enhanced Chemiluminescence (RPN2105, Amersham). Anti-ZMIZ1 (#89,500), anti-Beclin-1 (#3738), and anti-SIRT1 (#2310) were purchased from Cell Signaling Technology. Anti-β-actin (ab8226), anti-ATG5 (ab108327), anti-ATG7 (ab52472), anti-BNIP3 (ab109362), anti-P62 (ab109012), anti-LC3 (ab192890), and anti-FOXO3a (ab23683) were obtained from Abcam.

### EdU Stain

EdU can replace thymidine for insertion into the DNA molecules that are replicating during cell proliferation, allowing efficient detection of the percentage of cells in S phase. The Cell-Light EdU Apollo567 In Vitro Kit (C10310-1, RIBOBIO) was used according to the manufacturer's product instructions. Briefly, cells were treated with50 μM of EdU at 37 °C for 2 h. After EdU labeling of cells was completed, cells were fixed with 4% formaldehyde for 15 min at room temperature, followed by 2 mg/mL of glycine solution to neutralize the paraformaldehyde, and incubated with 0.5% Triton X-100 for 20 min at room temperature to enhance fixation. Apollo567 staining reaction solution was used to incubate cells for 30 min in a light-avoidant environment to react with EdU. Finally, 5 μg/mL DAPI staining solution was used to label the nuclei by incubating the cells for 15 min under light protection. Immediately after the staining was completed, it was detected using a fluorescence microscope.

### Cell-Counting Kit-8 (CCK-8) Assay

5 × 10^3^ cells were seeded in one well of a 96-well plate. After 6 h of incubation, the cells were adhered to the wall (recorded as 0 h at this point). Thereafter, cells were assayed every 24 h of incubation. The culture medium was discarded, cells were washed with PBS, and 10 μL CCK-8 reagent (PF00004, PTG) and 90 μL serum-free medium were added into, followed by incubation in an incubator for 2 h. Subsequently, the absorbance values of each well at 450 nm were measured using a microplate reader.

### Flow Cytometry for Apoptosis

Apoptosis was detected using the Annexin V-FITC/PI Kit (FXP018-100, 4A BIOTECH) according to the manufacturer's instructions. Briefly, cells were digested and harvested with EDTA-free trypsin (KGM25200, KeyGEN BioTECH), and resuspended in 1 × binding buffer to adjust cell density to 1 × 10^6^/ml. 5 µl of Annexin V/FITC was added into 100 µl of cell suspension that were incubated for 5 min at room temperature in the dark. Then, 10 µl of PI staining solution and 400 µl of PBS were added to the mixture that gently shaken and immediately assayed on the machine.

### Real-Time Fluorescence Quantitative PCR (RT-qPCR)

Cells were lysed using Trizol reagent, and total cellular RNA was extracted using RNA Extraction Kit (CW0581S, CWBIO). RNA was reversed to cDNA using the PrimeScript RT Master Mix (CW2569M, CWBIO). RT-PCR was performed with an SYBR Green master mix reagent (CW0957M, CWBIO). Β-actin was used as an internal reference, and relative gene expression was calculated using the 2^−ΔΔCT^ method. The primer sequences used were designed and synthesized by ORIGENE as follows: ZMIZ1 F 5’-GTCAGCAACCATGTGTTCCACC-3’, R 5’-GCCAGTTGGTGTTCATCTGCCG-3’; SIRT1 F 5’-TAGACACGCTGGAACAGGTTGC-3’, R 5’-CTCCTCGTACAGCTTCACAGTC-3’; β-actin F 5’-CACCATTGGCAATGAGCGGTTC-3’, R 5’-AGGTCTTTGCGGATGTCCACGT-3’.

### Co-immunoprecipitation

The pcDNA3.1-SIRT1 (G112237, NM_001142498, transcript variant 2) and pcDNA3.1-ZIMI1 (G156330, NM_020338) expression vectors were purchased from YouBio. Expression vectors were co-transfected into cells using Lipofectamine 2000 and transfected cells were treated with MG132 (HY-13259, MedChemExpress). After 24 h, cells were lysed with pre-cooled RIPA lysis buffer containing protease inhibitors (CW2200S, CWBIO) for 10 min on ice. Cell lysates were centrifuged at 12,000*g* for 15 min at 4 °C to collect the protein supernatant. 1 mg of protein solution was incubated with 2 µg of antibody (anti-ZMIZ1, anti-SIRT1, or anti-FOXO3a) overnight at 4 °C. Subsequently, the antigen–antibody mixture and 30 μl of resuspended protein A-agarose were incubated overnight at 4 °C. Transient centrifugation at 12,000*g* for 5 s was performed to collect the agarose magnetic bead-antigen–antibody complexes and washed with pre-cooled PBS. The complexes were boiled and centrifuged for separation of the components. Precipitated proteins were analyzed by western blot using antibody, including anti-ZMIZ1, anti-SIRT1 and anti-acetylated lysine (#9441, Cell Signaling Technology).

### Statistical Analysis

The results of three independent replicate experiments were analyzed using SPSS 22.0 software, and presented as mean ± SD. Student's *t*-test was used to compare the differences between the two groups, and *P* < 0.05 was considered a statistically significant difference.

## Results

### Knockdown of ZMIZ1 Inhibits Autophagy and Proliferation, and Induces Apoptosis of Colon Cancer Cells

**Fig. 1:**
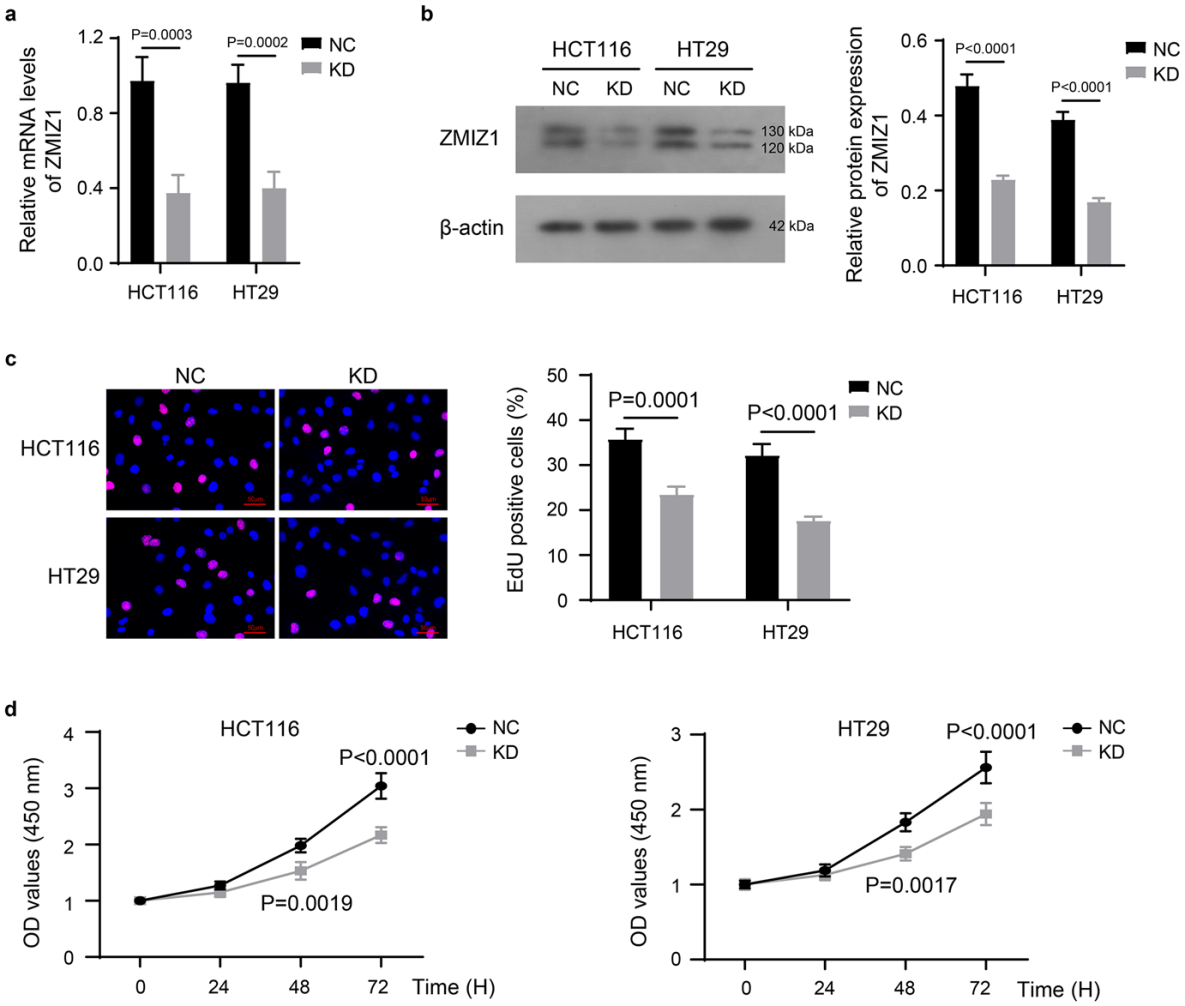
Knockdown of ZMIZ1 inhibits proliferation of colon cancer cells. The mRNA (**a**) and protein (**b**) levels of ZMIZ1 in HCT116 and HT29 cells which were transfected with specific shRNA targeting ZMIZ1 (KD). And, the proliferation of HCT116 and HT29 cells were measured with EdU (**c**) and CCK-8 (**d**) stain. **c** scale bars, 50 µm

Firstly, we knocked down the expression of ZMIZ1 in HCT116 and HT29 cells using specific shRNA transfection. The results of RT-qPCR (Fig. 1a) and western blot (Fig. 1b) confirmed that after shRNA targeting ZMIZ1 transfection (KD group), the intracellular levels of both ZMIZ1 mRNA and protein were significantly decreased in HCT116 and HT29 cells.

Subsequently, EdU staining showed that the proportion of EdU-positive cells was significantly reduced in the KD group compared with the NC group (Fig. 1c). EdU-positive cells are cells undergoing DNA replication that can undergo further cell division and proliferation. Furthermore, the results of CCK-8 assay indicated that the KD group showed a significant decrease in the absorbance value at 450 nm after staining with CCK-8 reagent (Fig. 1d). The absorbance value was positively correlated with the number of living cells. The results of both experiments indicated that ZMIZ1 knockdown significantly the proliferation of HCT116 and HT29 cells.

**Fig. 2:**
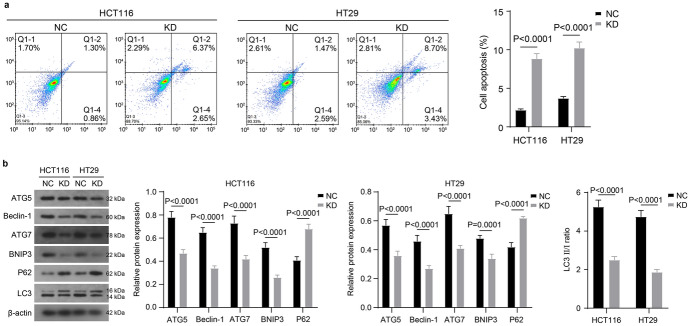
Knockdown of ZMIZ1 inhibits autophagy and induces apoptosis of colon cancer cells. HCT116 and HT29 cells were transfected with specific shRNA targeting ZMIZ1 (KD). And, **a** the apoptosis was detected with flow cytometry, **b** the expression of autophagy marker proteins was measured with western blot

Next, we examined the PI and Annexin V staining status of both groups of cells using flow cytometry. In the presented data, depending on the staining status, the Q1 quadrant (Annexin V−PI+) were necrotic cells and contained mechanically damaged cells. The Q2 quadrant (Annexin V+PI+) were late apoptotic cells, the Q4 quadrant (Annexin V+PI−) were early apoptotic cells, and the Q3 quadrant (Annexin V−PI−) were live cells. We counted the proportion of apoptotic cells in both groups by treating cells in the Q2 and Q4 quadrants as apoptotic cells. We found that the proportion of apoptotic cells was significantly increased in the KD group compared with the NC group (Fig. 2a). This results indicated that ZMIZ1 knockdown significantly induced apoptosis in HCT116 and HT29 cells.

Most interestingly, we found that ZMIZ1 was involved in the regulation of autophagy in HCT116 and HT29 cells. Beclin-1 is involved in the initiation of autophagy and stimulates the formation of phagocytic vesicles. ATG5, ATG7, and LC3 are the key proteins involved in the extension of autophagic vesicles and the formation of the autophagosome. The role of BNIP3 is to ensure that mitochondrial autophagy occurs. They are all key proteins indispensable to the autophagy process and are marker proteins for cellular autophagy. And P62 is involved in the ubiquitination degradation of LC3, and its expression is negatively correlated with autophagic activity. We detected the expression of these cellular autophagy marker proteins using western blot. The results revealed that the expression of autophagy marker proteins (ATG5, Beclin-1, ATG7, BNIP3 and LC3) was significantly reduced in cells of the KD group compared with the NC group, while the expression of p62, a negative regulator of autophagy, was significantly increased (Fig. 2b). This result indicated that ZMIZ1 knockdown significantly inhibited the autophagy of HCT116 and HT29 cells.

### ZMIZ1 Mediates Ubiquitination Degradation of SIRT1

**Fig. 3:**
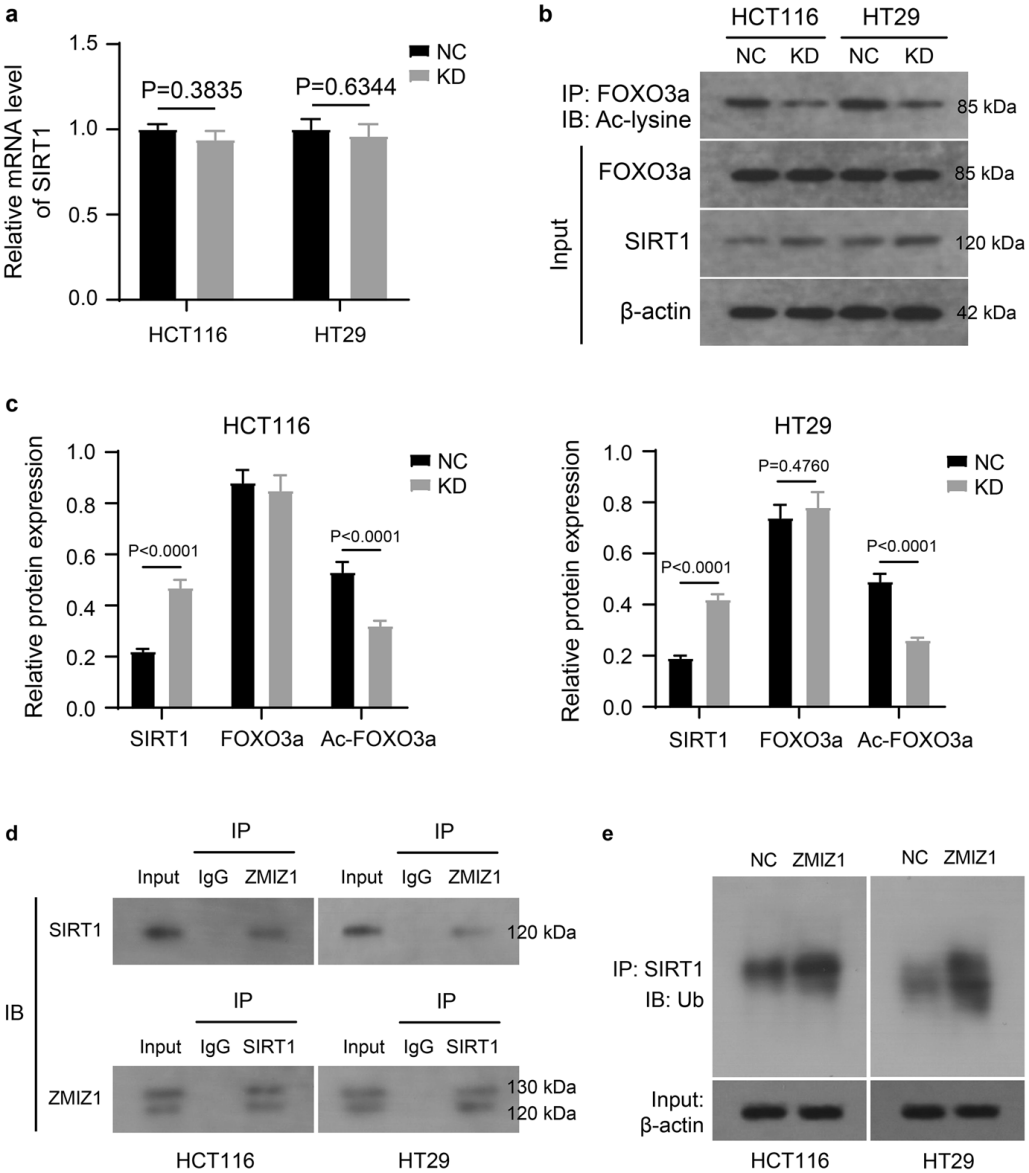
ZMIZ1 mediates ubiquitin–proteasome degradation of SIRT1. The mRNA (**a**) and protein (**b** and **c**) levels of SIRT1 in HCT116 and HT29 cells which were transfected with specific shRNA targeting ZMIZ1 (KD). The binding of SIRT1 and ZMIZ1 (**d**), and ubiquitinated SIRT1 levels (**e**) in HCT116 and HT29 cells which were overexpressed SIRT1 and ZMIZ1, and treated with MG132

In view of the above results, we wanted to further explore the major molecular mechanisms by which ZMIZ1 regulated the proliferation, autophagy and apoptosis of colon cancer cells. Therefore, we used the UbiBrowser 2.0 website to predict that ZMIZ1 could act as an E3 ubiquitin ligase and mediate the ubiquitination degradation of SIRT1. The results of RT-qPCR suggested that ZMIZ1 knockdown had no effect on the mRNA level of SIRT1 in HCT116 and HT29 cells (Fig. 3a). However, the results of western blot showed that knockdown of ZMIZ1 significantly reduced the protein level of SIRT1 in the cells (Fig. 3b and c). This result suggested that ZMIZ1 may be involved in the post-transcriptional modification of SIRT1.

In view of the possible role of ZMIZ1 as an E3 ubiquitin ligase, we subjected HCT116 and HT29 cells to the treatment of the proteasome inhibitor MG132 to preserve and more easily detect the ubiquitination status of SIRT1 as well as the possible role with ZMIZ1. We first immunoprecipitated ZMIZ1 and proteins bound to ZMIZ1 from HCT116 and HT29 cells (MG132-added) with anti-ZMIZ1 antibody, and subsequently, we achieved immunoblotting SIRT1 from proteins bound to ZMIZ1 (Fig. 3d top). Consistent with this logic, we also immunoblotted to ZMIZ1 from proteins bound to SIRT1 (Fig. 3d bottom). This result demonstrated the mutual binding of SIRT1 and ZMIZ1 in HCT116 and HT29 cells. Furthermore, also using immunoprecipitation experiments, we identified a significant increase in intracellular ubiquitinated SIRT1 after ZMIZ1 overexpression (MG132-added) (Fig. 3e). Taken together, these data suggested that ZMIZ1 can bind to SIRT1 and mediate its ubiquitinated degradation.

FOXO3a is a specific substrate for SIRT1, which regulates the transcription activity of FOXO3a on target genes by deacetylating FOXO3a (Jeung et al. 2016). And FOXO3a is a key balancer linking autophagy and apoptosis through transcriptional regulation of target genes (Fitzwalter & Thorburn 2018). As shown in Fig. 3b and c, there was no significant change in the intracellular FOXO3a protein level after ZMIZ1 knockdown, but the level of acetylated FOXO3a was significantly reduced. ZMIZ1 may further affect the deacetylation of FOXO3a through the regulation of SIRT1 protein stability.

### ZMIZ1 Regulates Autophagy, Proliferation and Apoptosis of Colon Cancer Cells Through the SIRT1/FOXO3a Axis

**Fig. 4:**
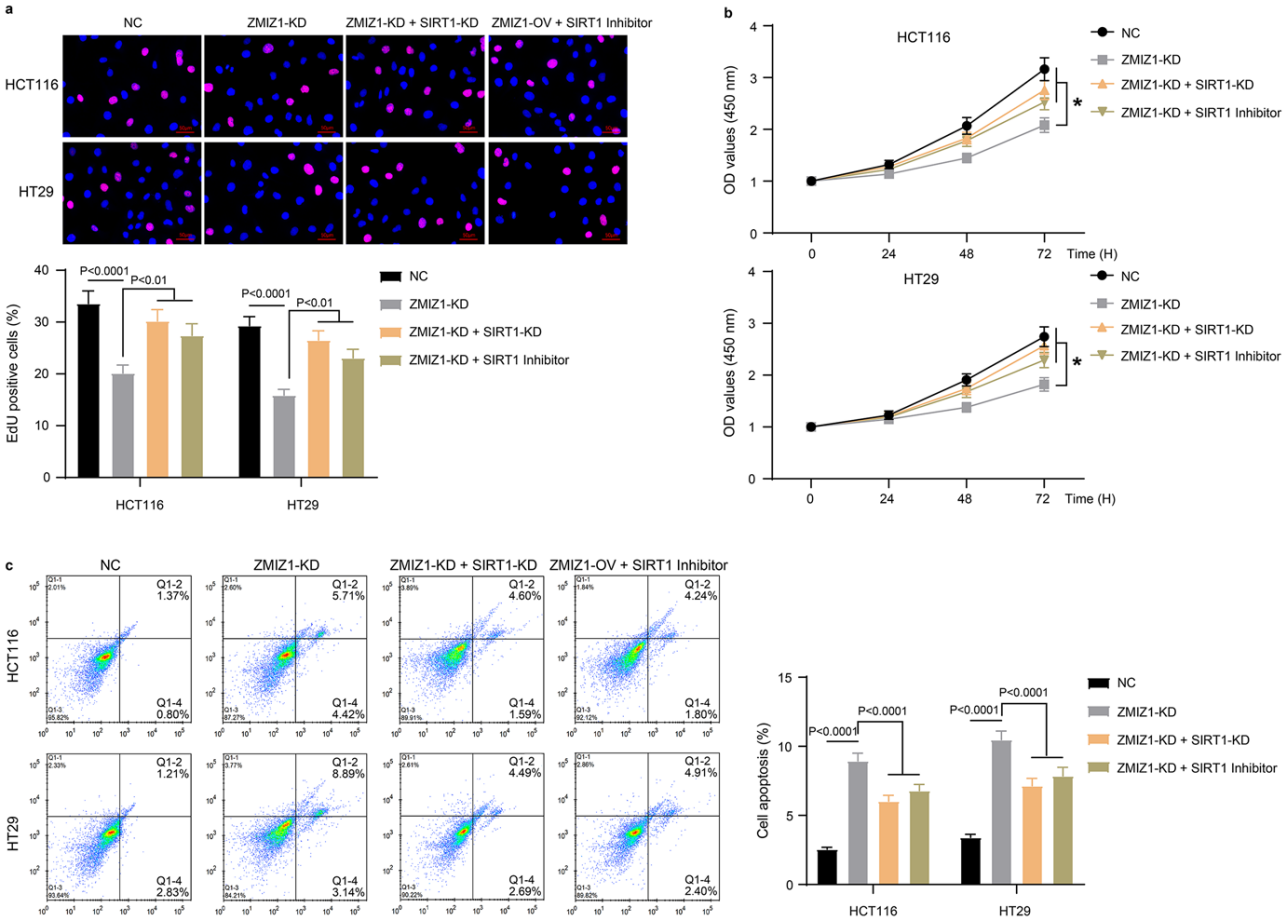
ZMIZ1 regulates proliferation and apoptosis of colon cancer cells via SIRT1. HCT116 and HT29 cells that were transfected with specific shRNA targeting ZMIZ1 (ZMIZ1-KD) were transfected with specific shRNA targeting SIRT1 (SIRT1-KD) or treated with SIRT1 Inhibitor. And, the proliferation of HCT116 and HT29 cells were measured with EdU (**a**) and CCK-8 (**b**) stain, (**c**) the apoptosis was detected with flow cytometry. *P < 0.05

**Fig. 5:**
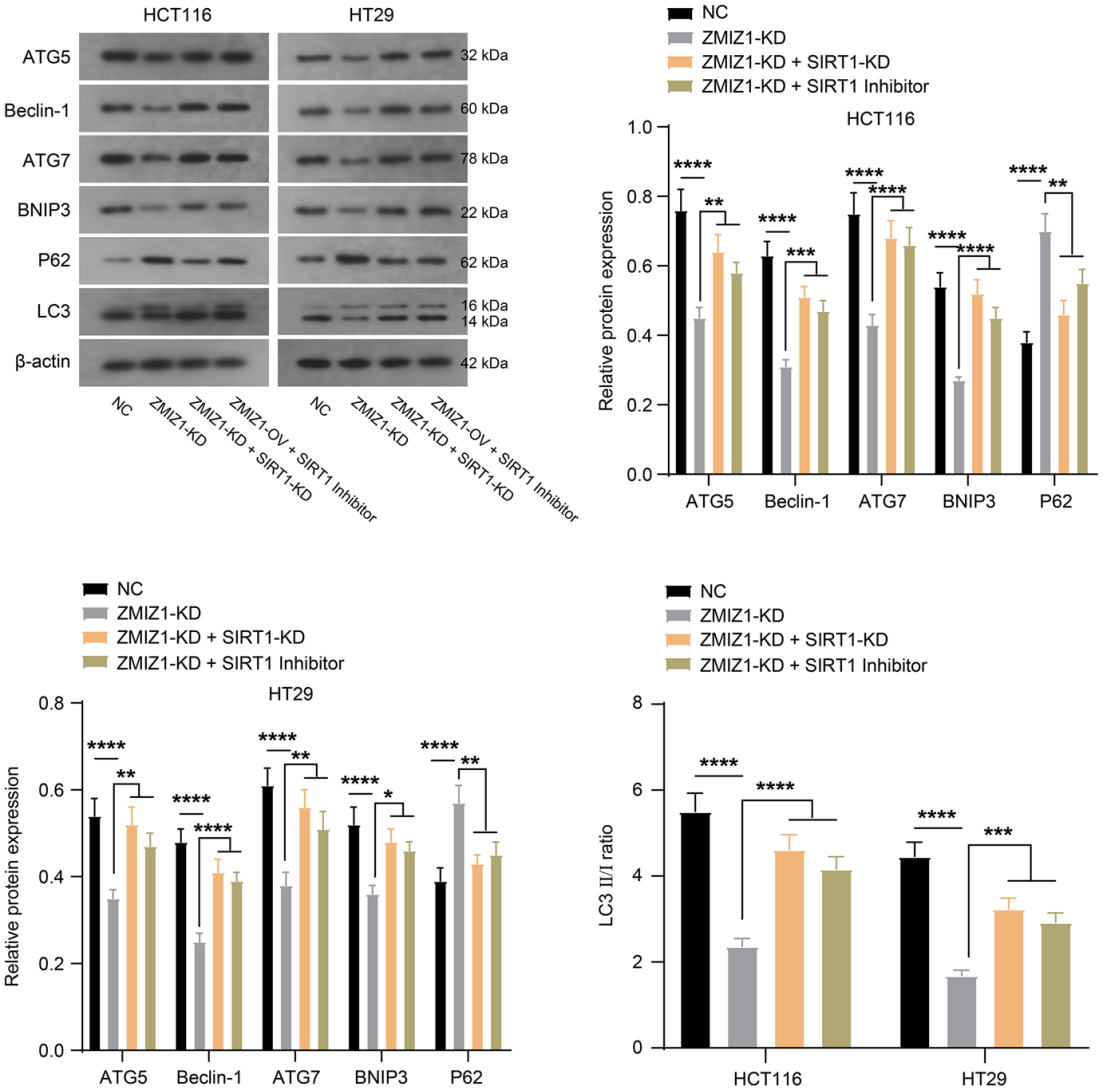
ZMIZ1 regulates autophagy of colon cancer cells via SIRT1. HCT116 and HT29 cells that were transfected with specific shRNA targeting ZMIZ1 (ZMIZ1-KD) were transfected with specific shRNA targeting SIRT1 (SIRT1-KD) or treated with SIRT1 Inhibitor. And, the expression of autophagy marker proteins was measured with western blot. *P < 0.05, **P < 0.01, ***P < 0.001, ****P < 0.0001

To clarify whether ZMIZ regulates autophagy, proliferation and apoptosis of colon cancer cells through SIRT1, we set up two rescue experiments in which the cells of ZMIZ1 knockdown (ZMIZ-KD) were subjected to SIRT1 knockdown (SIRT-KD) or pharmacological inhibition (SIRT1 Inhibitor) (EX527, 100 nM). Knockdown of ZMIZ1 inhibited the proliferation of HCT116 and HT29 cells, whereas both SIRT1 knockdown and pharmacological inhibition compensated for the inhibitory effect of ZMIZ1 knockdown on cell proliferation (Fig. 4a and b). Knockdown of ZMIZ1-induced apoptosis in HCT116 and HT29 cells, whereas both SIRT1 knockdown and pharmacological inhibition impeded the apoptotic induction by ZMIZ1 knockdown (Fig. 4c). Finally, ZMIZ1 knockdown affected the expression of key proteins in the autophagy process and inhibited cellular autophagy. In contrast, both SIRT1 knockdown and pharmacological inhibition neutralized the inhibitory effect of ZMIZ1 knockdown on cellular autophagy (Fig. 5). These data suggested that ZMIZ1 indeed regulates the fate decision of colon cancer cells through SIRT1.

## Discussion

Several recent population-based cohort studies have indicated that differences in ZMIZ1 expression due to gene mutations or epigenetic regulatory mechanism are significantly associated with the survival of cancer patients, including those with colon cancer (Arnau-Collell et al. 2020; Mathios et al. 2019; Song et al. 2017; Zhang et al. 2014). All of these studies have demonstrated the critical role of the ZMIZ1 protein in colon cancer and in cancer progression. However, to the best of our knowledge, no experiments have been performed to confirm or validate the specific role of ZMIZ1 in colon cancer progression. The present study is the first of its kind to investigate the knockdown of ZMIZ1 in colon cancer cell lines in vitro using specific shRNA transfection, and found that ZMIZ1 knockdown significantly inhibited proliferation and autophagy and induced apoptosis of cancer cells. ZMIZ1 plays a key pro-cancer role in colon cancer. Using in vitro cellular and loss-of-function assays, this study demonstrated that ZMIZ1 plays a critical pro-cancer role in colon cancer and that targeting ZMIZ1 is beneficial in hindering colon cancer progression. Our results are consistent with the trend of previous cohort findings that ZMIZ1 is significantly overexpressed in colon cancer tissues and predicts a poor prognosis for patients (Mathios et al. 2019). Unfortunately, only in vitro cellular experiments were performed in the present study, which failed to be further functionally validated in animal models or analyzed in further expanded clinical population samples.

In addition to colon cancer, a few studies have been reported on the molecular function of ZMIZ1 in other types of solid tumors. Mathios et al. find that ZMIZ1 significantly promotes the migration of glioblastoma cell line (U87), but has no effect on cell proliferation and apoptosis (Mathios et al. 2019). Wang et al*.* report that hepatocyte-specific *ZMIZ1* knockout mice exhibit significant hindrance of intrahepatic cholangiocarcinoma formation (Wang et al. 2022). Rogers et al*.* suggest that ectopic expression of ZMIZ1 induces cutaneous squamous cell malignancy in a mouse model of cancer (Rogers et al. 2013). In conclusion, even in different types of solid tumors, ZMIZ1 exerts significant pro-carcinogenic effects and even drives carcinogenesis, and its targeting would be beneficial in hindering cancer. However, the reasons for its varying degrees of effects on a wide range of cancer cell behaviors, including proliferation, autophagy, apoptosis, and migration, remain to be explored and may depend on the overall state of the cancer cell, or be influenced by other signals or regulated by other mechanisms. Overall, ZMIZ1 plays a critical role in cancer initiation and progression, but studies on its specific molecular functions and regulatory mechanisms are still in their infancy.

The present study elucidates for the first time the possible molecular function of ZMIZ1 as an E3 ubiquitin ligase that mediated the ubiquitination-proteasomal degradation of SIRT1. In previous studies, ZMIZ1 is mainly annotated as a transcriptional co-activator that enhances the transcriptional activity of transcription factors, including p53, the androgen receptor (AR), and Smad3 (Huang et al. 2005; Lee et al. 2007; Li et al. 2006; Sharma et al. 2003). Moreover, ZMIZ1 enhances the transcriptional activity of AR by promoting the sumoylation of AR (Sharma et al. 2003). Whereas, the enhancement of the transcriptional activity of p53 by ZMIZ1 is thought to be a SUMO-independent pathway (Lee et al. 2007). Sumoylation reaction is a class of ubiquitination-like reactions that mediates the subcellular localization and transcriptional activity of substrates. The sumoylation by ZMIZ1 is thought to be mediated by its highly conserved SP-RING structural domain, which is utilized to act as SUMO E3 ligase that binds SUMO and SUMO-binding enzyme UBC9 and facilitates SUMO-binding to specific substrates (Rytinki et al. 2009; Schmidt & Muller 2003). In this study, we successively examined the effects of ZMIZ1 knockdown on SIRT1 mRNA and protein levels, the direct binding of ZMIZ1 to SIRT1, and the effects of ZIMZ1 exogenous expression on the ubiquitination-modification status of SIRT1. The experimental data obtained fully demonstrated that ZMIZ1 acts as an E3 ubiquitin ligase and mediates the ubiquitination-proteasomal degradation of SIRT1. This is a novel molecular function of ZMIZ1 that has never been reported before. In addition, it has been hypothesized that ZMIZ1 may play an important role in chromatin assembly and chromatin maintenance (Lee et al. 2007). And based on the histone deacetylation function of SIRT1, we also proposed the hypothesis that ZMIZ1 may regulate chromatin function by mediating the degradation of SIRT1. However, this study did not confirm whether ZMIZ1 transfers ubiquitin from E2 ubiquitin conjugating enzyme to the substrate SIRT1 via the SP-RING structural domain, and failed to explore the mechanism regulating the subcellular localization of ZMIZ1.

After subsequent rescue experiments, we also demonstrated that ZMIZ1 could promote the malignant proliferation of colon cancer by facilitating the degradation of SIRT1. Both knockdown and pharmacological inhibition of SIRT1 neutralized the effects of ZMIZ1 knockdown on cancer cells. Although the role of SIRT1 in colon cancer remains controversial. Of interest is the role of ZMIZ1 in autophagy of colon cancer cells. Autophagy is a cell survival mechanism induced by intracellular and extracellular stress that protects cancer cells from apoptosis (Fitzwalter & Thorburn 2015). Autophagy, apoptosis and other modes of cell death determine cell fate through crosstalk signals, and there are numerous molecules present in these crosstalk signals. *Sirt1* has also been reported to be an autophagy-related gene in colorectal cancer (Mo et al. 2019). Heterozygous deletion of the *Sirt1* gene enhances proliferation, autophagy, stress resistance, and cancer formation (Ren et al. 2017). In contrast, pure deletion of the *Sirt1* gene triggers the apoptotic pathway, increases cell death, and decreases autophagy and cancer formation (Ren et al. 2017). Intestine-specific *Sirt1* heterozygous mice have enhanced intestinal tumor formation, whereas intestine-specific *Sirt1* pure knockout mice have reduced colon cancer development (Ren et al. 2017). Among the action substrates of SIRT1, FOXO3a is particularly conspicuous due to its role in balancing autophagy and apoptosis (Fitzwalter & Thorburn 2018; Fitzwalter et al. 2018). When subjected to stress, FOXO3a can rapidly respond to trans-activate autophagy-related genes and induce autophagy in cells (Fitzwalter & Thorburn 2018; Fitzwalter et al. 2018). When autophagy is persistently blocked, FOXO3a can trans-activate apoptosis (Fitzwalter & Thorburn 2018; Fitzwalter et al. 2018). SIRT1/FOXO3a signaling plays an important role in the proper autophagy activation and protection of neuronal cells (Xie et al. 2022). Promoting FOXO3a-mediated autophagic flux may facilitate cancer formation (Zhu et al. 2021). SIRT1-mediated deacetylation of FOXO3a regulates the transcriptional activity of FOXO3a, as well as the proliferation and apoptosis of cancer cells (Jeung et al. 2016). The experimental data of the present study also suggested that ZMIZ1 affected the level of acetylated FOXO3a by mediating the degradation of SIRT1. We hypothesized that ZMIZ1 may control the fate of colon cancer cells through the SIRT1/FOXO3a axis.

In conclusion, ZMIZ1 significantly promoted the malignant proliferation of colon cancer cells, possibly by acting as an E3 ubiquitin ligase that regulates the SIRT1/FOXO3a axis, thereby controlling autophagy or apoptosis of colon cancer cells. Targeting ZMIZ1 would be beneficial for the treatment of colon cancer.

## Data Availability

The datasets used and/or analysed during the current study are available from the corresponding author on reasonable request.
